# Association of midlife body-weight variability and cycles with earlier dementia onset: a nationwide cohort study

**DOI:** 10.1186/s13195-024-01460-5

**Published:** 2024-04-25

**Authors:** Yujin Park, Su Hwan Kim, Jiwon Ryu, Hyung-Jin Yoon

**Affiliations:** 1 Healthcare Data Center, Kangbuk Samsung Hospital, Sungkyunkwan University School of Medicine, 29, Saemunan-ro, Jongno-gu, Seoul, 03181 Republic of Korea; 2 Department of Information Statistics, Gyeongsang National University, 501, Jinju-daero, Jinju-si, Gyeongsangnam-do 52828 Republic of Korea; 3 Hospital Medicine Center, Seoul National University Bundang Hospital, 83, Gumi-ro 173beon-gil, Bundang-gu, Seongnam-si, Gyeonggi-do 13620 Republic of Korea; 4 Department of Human Systems Medicine, Seoul National University College of Medicine, 103, Daehak-ro, Jongno-gu, Seoul, 03080 Republic of Korea; 5 Medical Big Data Research Center, Seoul National University Medical Research Center, Seoul National University College of Medicine, 103, Daehak-ro, Jongno-gu, Seoul, 03080 Republic of Korea

**Keywords:** Body weight change, Early dementia risk, Mid-life body weight change

## Abstract

**Background:**

Given the rising awareness of health-related lifestyle modifications, the impact of changes in body weight (BW) on cognitive function and dementia generates significant concern. This study aimed to investigate the association between BW changes and dementia in a middle-aged Korean population.

**Methods:**

A retrospective, population-based longitudinal study was conducted utilizing data from the National Health Insurance Service (NHIS) database. Participants aged 40 years or older in 2011 who underwent at least five health checkups between 2002 and 2011 were followed-up for dementia until 2020. A total of 3,635,988 dementia-free Korean aged < 65 at baseline were examined. We analyzed the association between BW variability independent of the mean (VIM) with BW cycle, defined as either an upward or a downward direction of BW, and the risk of incident dementia.

**Results:**

The results showed an increased risk of dementia in the highest quartile of VIM quartile (hazard ratio [HR] 1.52, 95% confidence interval [CI] 1.47–1.58) compared to the lowest quartile of VIM. Additionally, the results showed an even higher increased risk of dementia in the highest BW cycle (≥ 2 cycles of 10% BW = HR 2.00, 95% CI 1.74–1.29). Notably, the combined concept of VIM with BW cycle showed an even higher dementia risk (highest quartile of VIM with ≥ 2 cycles of 10% BW = HR 2.37, 95% CI 2.05–2.74) compared to the baseline group (lowest quartile of VIM with < 3% BW cycle).

**Conclusions:**

The present study highlights the importance of considering BW changes with BW variability along with the BW cycle to assess dementia risk in detail, providing valuable insights for preventive strategies.

**Supplementary Information:**

The online version contains supplementary material available at 10.1186/s13195-024-01460-5.

## Background

Body weight (BW) varies throughout an individual’s life. With increased health awareness, people have tried to modify their lifestyles to prevent several diseases. Repeated attempts at lifestyle modification lead people to experience BW fluctuations, which tend to increase or decrease rather than maintain a constant weight. Fluctuations in BW, also known as weight variability, are widely recognized to increase the risk of adverse health outcomes, such as metabolic syndrome, diabetes, hypertension, and cardiovascular disease [1–3]. Furthermore, fluctuation in BW could affect the cognitive function and dementia [4, 5].

Dementia is a major health problem that imposes a significant economic burden. Dementia has several modifiable risk factors, including high blood pressure, obesity, physical inactivity, and an unhealthy diet [6]. Obesity can affect brain health through the actions of adipocytes, adipocyte-associated hormones, and cytokines. Moreover, these substances may cross the blood-brain barrier and influence various brain health, including energy homeostasis, learning, and memory [7, 8]. However, even low body mass index (BMI) influences cognitive function and dementia risk [9, 10]. These findings suggest the importance of the BW trajectory rather than a focus on the BW at a specific time-point to provide more information regarding the risk of dementia. Several studies have investigated the association between BW variability and the risk of dementia, using various variability parameters, such as variability independent of the mean (VIM), coefficient of variation (CV), standard deviation (SD), and average successive variability (ASV) [4, 11]. These parameters indicate the range of fluctuation but do not specify the direction of fluctuation as either a decrease or an increase. Therefore, we introduced a new concept in BW variability, termed the “BW cycle,” defined as a change in BW followed by a subsequent change in the opposite direction [12]. We assessed the risk of dementia in both mid-life and late-life stages by using the BW cycle concept and compared the effectiveness of these assessments with that using only the VIM.

## Methods

### Study population

The National Health Insurance Service (NHIS) is a governmental single-insurance organization, and the NHIS provides health checkups, among other services. As health insurance members undergo health checkups every 1 or 2 years, the NHIS stores national health checkup data as well as claims data. The NHIS database contains an eligibility database (based on characteristics of age, sex, and socioeconomic status), a health checkup database (including common questionnaires on health-related habits, e.g., smoking status, alcohol intake, physical activity, medical history, and family history), measurement data (e.g., height, weight, and blood pressure), laboratory results (e.g., total cholesterol, fasting blood sugar, and serum creatinine), and a medical history database (e.g., diagnosis and medication). More detailed information regarding the health checkup database has been described previously [13]. 

**Fig. 1:**
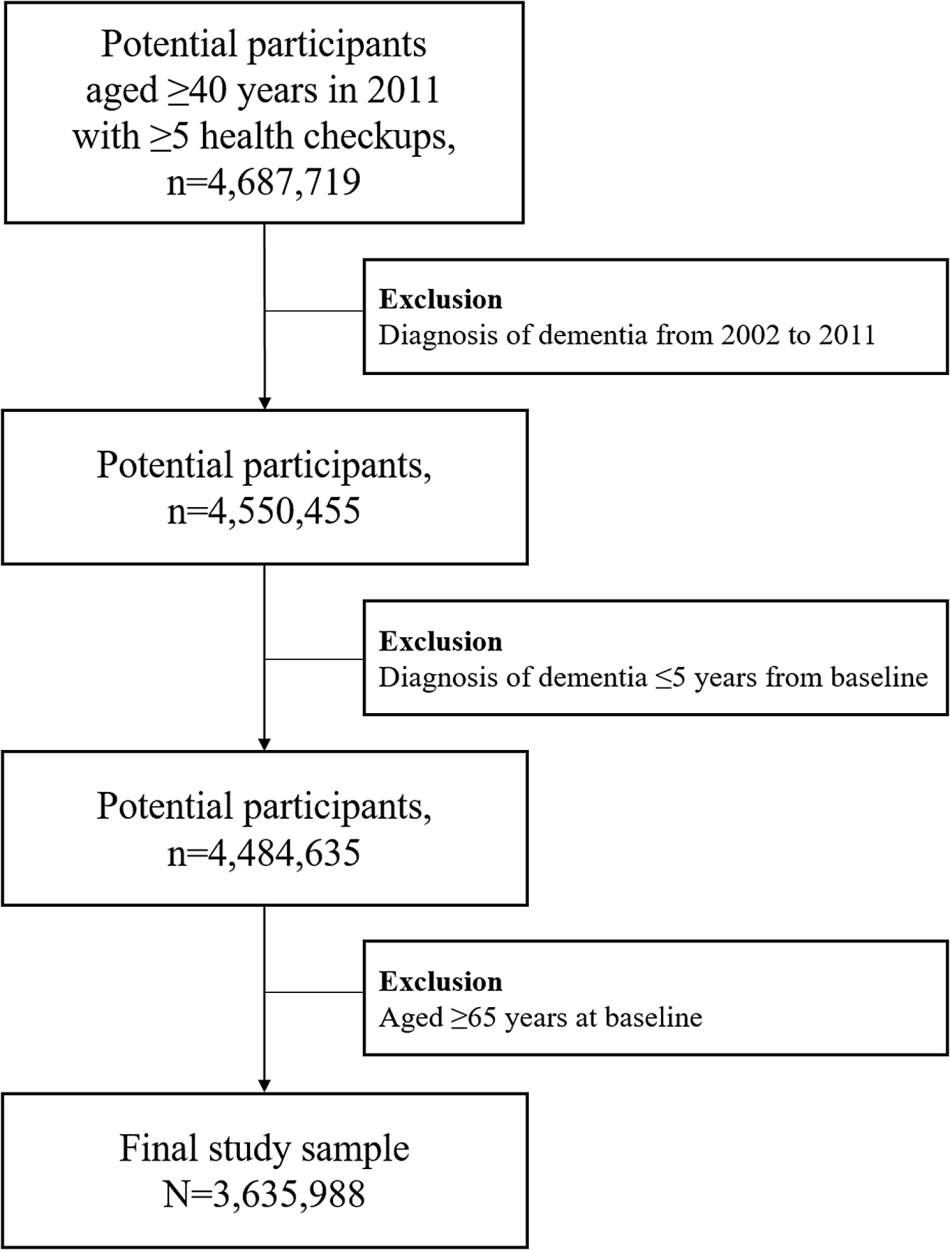
Flowchart of the study population

Data from participants aged ≥ 40 years in 2011 who underwent at least five health checkups between January 1, 2002, and December 2011 were screened and a total of 4,687,719 participants were included after the relevant exclusions. We excluded 137,264 participants with a previous diagnosis of dementia between 2002 and 2010/2011. To avoid the possibility of a time lag in the detection of dementia, we additionally excluded participants who were diagnosed with dementia within 5 years before the baseline (2010/2011). Participants were then followed up until the date of dementia onset or December 31, 2020, whichever came first. To achieve the objective of identifying BW variability in midlife and assess the risk of early dementia, we included participants with a baseline age < 65 years, which included 3,635,988 dementia-free middle-aged participants (Fig. 1). Our study focused on investigating how mid-life BW changes influence the risk of developing dementia before the age of 70 years. To assess this association, we included 3,635,988 dementia-free participants aged < 65 years.

This study was conducted in accordance with relevant guidelines and regulations. The Institutional Review Board of Seoul National University Hospital waived the need for informed consent for this study because the analyses used existing data and there was no contact with individuals (IRB waiver No: E-2311-055-1483).

### BW change

Intra-individual BW variability was assessed using variability independent of the mean (VIM) was k×standard deviation (SD)/meanT)^β^, where β was calculated from mean and SD using a fitting curve based on the model where SD = constant/mean^β^ [14, 15]. The BW cycle was evaluated with multiple thresholds (≥ 3%, ≥ 5%, ≥ 7%, and ≥ 10% of BW), defined as when BW changes in either an upward or a downward direction above a specified threshold percentage [12]. 

### Clinical definitions

The primary outcome of this study was newly diagnosed, physician-diagnosed dementia. Dementia was defined according to the International Classification of Diseases, 10th revision (ICD-10) codes: F00 (dementia in Alzheimer’s disease), F01 (vascular dementia), F02 (dementia in other diseases), F03 (unspecified dementia), F05 (senile dementia with delirium), and G30 (Alzheimer’s disease).

Information on health-related behaviors (smoking status, alcohol intake, and physical activity) and family history of hypertension (HTN), type 2 diabetes mellitus (DM), heart disease, and stroke was obtained from self-reported questionnaires. Smoking status was categorized as never smoker, ex-smoker, or current smoker. Alcohol intake were classified as none, < 10 g/day, 10–19.9 g/day, 20–39.9 g/day, and ≥ 40 g/day. Physical activity was defined using the long-form International Physical Activity Questionnaire (IPAQ), where individuals were classified based on metabolic equivalents (METS) value of < 600, 600–3000, and ≥ 3000 [16]. 

HTN was defined as systolic/diastolic blood pressure ≥ 140/90 mmHg or at least 3 months of medication. DM was defined as fasting blood sugar ≥ 126 or at least 3 months of medication. Further details of past medical history (HTN, DM, heart disease, and stroke) information was obtained from self-reported questionnaire.

### Statistical analysis

The participants were classified into 36 groups according to the BW variability (VIM quartiles) and BW cycles. Data were presented as mean ± SD for continuous variables and counts (%) for categorical variables.

Hazard ratios (HRs) and 95% confidence intervals (CI) for incident dementia in relation to the BW change were estimated using a Cox proportional hazards model with follow-up time as the timescale. In the current study, the follow-up time was calculated as the time from the participants’ last health checkup between 2010 and 2011 until dementia diagnosis or the last health checkup. The time of dementia diagnosis was defined as the first date in the claims history containing the diagnosis codes.

We considered age, sex, body mass index (BMI), systolic blood pressure (SBP), diastolic blood pressure (DBP), total cholesterol, estimated glomerular filtration rate (eGFR), health-related behaviors (smoking status, alcohol intake, and physical activity), medical history (HTN, DM, heart disease, and stroke), and family history (HTN, DM, heart disease, and stroke) to be potential risk parameters of dementia in Cox-models. Stratified analyses using BMI, sex, and medical history of HTN and DM were conducted to examine the effect of modification of the variables.

Two-tailed *P*-values < 0.05 were considered statistically significant. All statistical analyses were performed using SAS version 9.4 (SAS Institute, Cary, NC, USA) and R version 4.0.3 (R Core Team, Vienna, Austria).

## Results

### Baseline characteristics

The baseline characteristics of the participants with incident dementia are presented in Table 1. A total of 3,635,988 dementia-free participants were included in the study cohort, of 33.64% were female respondents. The mean age of participants was 51.14 (± 6.54) years, and the mean age at onset of dementia was 66.23 (± 4.89) years. During the mean follow-up duration of 9.67 (± 0.59) years, 21,060 dementia cases (48.45% in females) were observed. Compared with participants without dementia, those with incident dementia were older and more likely to have HTN, DM, heart disease, and stroke.

**Table 1:** Baseline characteristics of the study cohort

		Total (*N* = 3,635,988)	Non–dementia (*N* = 3,614,928)	Dementia (*N* = 21,060)
Age, years		51.1 ± 6.54	51.10 ± 6.53	58.55 ± 4.65
Female (%)		1,223,013 (33.64)	1,212,809 (33.55)	10,204 (48.45)
BMI		24.01 ± 2.91	24.01 ± 2.91	23.96 ± 2.97
SBP		122.77 ± 14.09	122.76 ± 14.09	124.62 ± 15.18
DBP		77.24 ± 9.85	77.24 ± 9.85	77.44 ± 10.01
Total cholesterol		198.87 ± 35.91	198.87 ± 35.90	198.60 ± 38.55
eGFR		88.26 ± 14.63	88.28 ± 14.62	84.85 ± 14.84
Health–related behaviors				
Smoking status (%)				
	Never smoker	1,887,224 (51.99)	1,873,761 (51.92)	13,463 (64.02)
	Ex–smoker	806,122 (22.21)	802,752 (22.24)	3,370 (16.02)
	Current Smoker	934,858 (25.81)	932,660 (25.84)	4,198 (19.96)
Alcohol intake (%)				
	None	1,674,203 (46.05)	1,661,091 (45.95)	13,112 (62.26)
	< 10 g/day	924,726 (25.43)	921,090 (25.48)	3,636 (17.26)
	10–19.9 g/day	478,530 (13.16)	476,781 (13.19)	1,749 (8.30)
	20–39.9 g/day	411,834 (11.33)	410,172 (11.35)	1,662 (7.89)
	≥ 40 g/day	146,695 (4.03)	145,794 (4.03)	901 (4.28)
Physical activity (METS) (%)				
	Low (< 600)	1,197,822 (32.94)	1,189,512 (32.91)	8,310 (39.46)
	Moderate (600–3000)	1,993,797 (54.84)	1,983,758 (54.88)	10,039 (47.67)
	High (≥ 3000)	444,369 (12.22)	441,658 (12.22)	2,711 (12.87)
Medical history (%)				
HTN		2,030,032 (55.83)	2,015,379 (55.75)	14,653 (69.58)
DM		918,510 (25.26)	911,053 (25.20)	7,457 (35.41)
Heart disease		50,277 (1.38)	49,553 (1.37)	724 (3.44)
Stroke		17,692 (0.49)	17,224 (0.48)	468 (2.22)
Family history (%)				
HTN		1,387,633 (38.16)	1,380,319 (38.18)	7,314 (34.73)
DM		930,071 (25.58)	925,056 (25.59)	5,015 (23.81)
Heart disease		476,735 (13.11)	474,388 (13.12)	2,347 (11.14)
Stroke		733,078 (20.16)	728,572 (20.15)	4,506 (21.40)

### Risk of incident dementia according to BW change

**Fig. 2:**
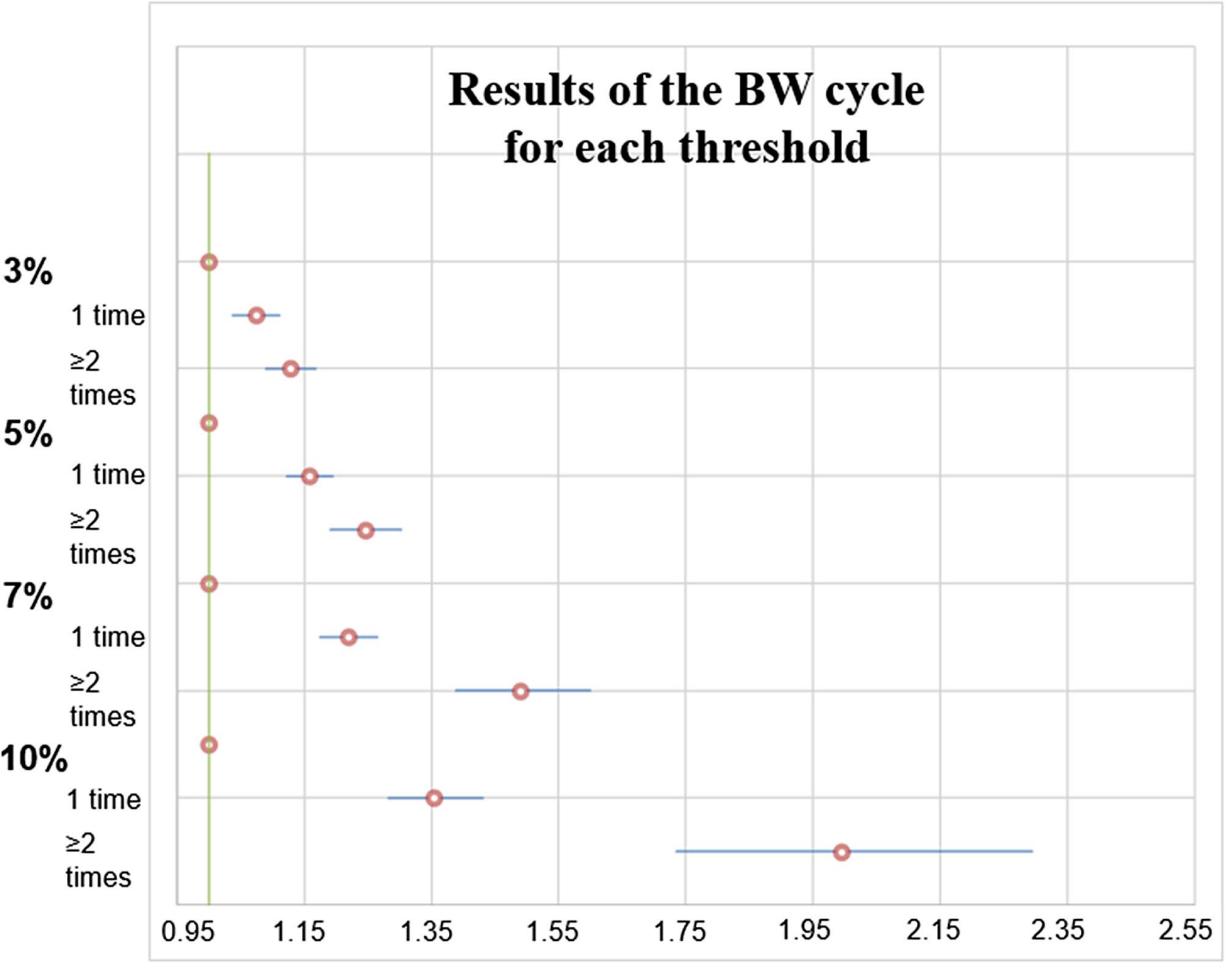
Association between body weight (BW) cycles and the risk of dementia

Table 2 presents the individual and combined effects of the association of BW cycle and BW variability (VIM quartiles) with incident dementia in the multivariate-adjusted model. For the individual effects, we found that the higher the threshold % and more of BW cycles were mostly associated with higher risk of dementia (Fig. 2). Furthermore, a dose effect was observed wherein the association strengthened with the degree and threshold of BW cycles. The HRs of BW cycling for incident dementia ranged from 1.07 (95% CI: 1.04–1.11) at one cycle of 3% BW to 1.13 (1.09–1.17) at ≥ 2 cycles of 3% BW. The HRs reached up to 1.35 (1.28–1.43) and 2.00 (1.74–2.29) at one cycle and ≥ 2 cycles of 10% BW, respectively. A similar trend was observed with BW variability (VIM quartiles) only results, in that higher variability was associated with a higher incidence of dementia. Compared to the reference group, the lowest quartile of VIM, participants in the highest quartile of VIM had an increased risk of dementia (HR 1.52; 95% CI 1.47–1.58). For the combined effects of the association between BW variability (VIM quartiles) with BW cycle and incident dementia, participants in the high BW variability group (highest quartile VIM with ≥ 2 cycles of 10% BW) had a significantly higher risk of incident dementia (2.37; 2.05–2.74) compared to the reference BW variability group (lowest quartile VIM with < 3% BW cycle).

**Table 2:** Individual and combined effects of the association of BW cycle and BW variability (VIM quartile) with incident dementia (Data are hazard ratios with 95% CI.)

		BW cycle only	VIM Q1	VIM Q2	VIM Q3	VIM Q4
VIM only		–	1.0 (ref)	**1.08 (1.04–1.12)**	**1.23 (1.18–1.28)**	**1.52 (1.47–1.58)**
< 3% (*N* = 1,166,108)		1.0 (ref)	1.0 (ref)	**1.10 (1.03–1.18)**	**1.15 (1.07–1.23)**	**1.46 (1.37–1.56)**
3%	1 time (*N* = 1,250,801)	**1.07 (1.04–1.11)**	1.03 (0.96–1.10)	1.03 (0.96–1.11)	**1.23 (1.13–1.33)**	**1.40 (1.28–1.53)**
	≥ 2 times (*N* = 1,219,079)	**1.13 (1.09–1.17)**	0.93 (0.84–1.03)	**1.13 (1.04–1.23)**	**1.28 (1.16–1.40)**	**1.35 (1.22–1.50)**
5%	1 time (*N* = 969,925)	**1.16 (1.12–1.20)**	0.91 (0.78–1.07)	**1.09 (1.02–1.17)**	**1.28 (1.19–1.37)**	**1.51 (1.39–1.63)**
	≥ 2 times (*N* = 414,193)	**1.25 (1.19–1.30)**	1.20 (0.68–2.12)	0.98 (0.85–1.13)	**1.26 (1.12–1.40)**	**1.57 (1.38–1.77)**
7%	1 time (*N* = 516,383)	**1.22 (1.18–1.27)**	None	0.97 (0.82–1.14)	**1.27 (1.18–1.36)**	**1.48 (1.38–1.59)**
	≥ 2 times (*N* = 115,333)	**1.49 (1.39–1.60)**	None	1.02 (0.38–2.71)	**1.29 (1.07–1.56)**	**1.69 (1.48–1.93)**
10%	1 time (*N* = 183,968)	**1.35 (1.28–1.43)**	None	None	1.08 (0.85–1.37)	**1.64 (1.53–1.75)**
	≥ 2 times (*N* = 21,385)	**2.00 (1.74–2.29)**	None	None	None	**2.37 (2.05–2.74)**

BW, body weight; VIM, variability independent of the mean; Q, quartile

Bolds are statistically significant

Table 3 shows the association between BW change and dementia risk in BMI-stratified subgroups. Higher adjusted HRs for dementia were observed for quartile of VIM and BW cycle in the group with BMI < 25 kg/m^2^ than in the group with BMI ≥ 25 kg/m^2^. In the case of the group with one cycle of 5% BW in BMI < 25 kg/m^2^ for instance, the risk of dementia increases gradually from VIM Q1 to VIM Q4 (0.77; 0.61–0.96, 1.13; 1.03–1.23, 1.29; 1.18–1.41, and 1.54; 1.40–1.70, respectively). As for VIM Q4 group in BMI < 25 kg/m^2^, furthermore, compared to those of < 3% BW cycle (1.52; 1.40–1.65), individuals with ≥ 2 cycles of 10% BW had far higher risk (2.48; 2.08–2.95).

**Table 3:** The BMI-stratified individual and combined effects of the associations of BW cycle and BW variability (VIM) with incident dementia (Data are hazard ratios with 95% CI.)

		BMI < 25 kg/m^2^ (*N* = 2,356,669)					BMI ≥ 25 kg/m^2^ (*N* = 1,279,319)				
		BW cycle only	VIM Q1	VIM Q2	VIM Q3	VIM Q4	BW cycle only	VIM Q2	VIM Q1	VIM Q2	VIM Q3
VIM only		–	1.0 (ref)	**1.09** **(1.03–1.15)**	**1.26** **(1.20–1.32)**	**1.57** **(1.50–1.65)**	–	1.0 (ref)	**1.07** **(1.00–1.14)**	**1.20** **(1.12–1.28)**	**1.45** **(1.36–1.54)**
< 3% (*N* = 1,166,108)		1.0 (ref)	1.0 (ref)	**1.12** **(1.03–1.22)**	**1.17** **(1.07–1.27)**	**1.52** **(1.40–1.65)**	1.0 (ref)	1.0 (ref)	1.08 (0.97–1.20)	1.11 (0.99–1.24)	**1.38** **(1.23–1.54)**
3%	1 time (*N* = 1,250,801)	**1.07** **(1.03–1.12)**	1.06 (0.97–1.15)	1.02 (0.93–1.12)	**1.31** **(1.19–1.45)**	**1.41** **(1.26–1.57)**	**1.07** **(1.02–1.13)**	0.99 (0.89–1.10)	1.04 (0.92–1.16)	1.07 (0.93–1.23)	**1.07** **(1.02–1.13)**
	≥ 2 times (*N* = 1,219,079)	**1.12** **(1.07–1.17)**	0.92 (0.81–1.04)	1.10 (0.99–1.222	**1.35** **(1.21–1.51)**	**1.40** **(1.24–1.59)**	**1.14** **(1.08–1.21)**	0.94 (0.79–1.12)	**1.19** **(1.03–1.37)**	1.13 (0.95–1.34)	**1.14** **(1.08–1.21)**
5%	1 time (*N* = 969,925)	**1.16** **(1.11–1.20)**	**0.77** **(0.61–0.96)**	**1.13** **(1.03–1.23)**	**1.29** **(1.18–1.41)**	**1.54** **(1.40–1.70)**	**1.17** **(1.11–1.23)**	1.18 (0.93–1.49)	1.05 (0.93–1.18)	**1.26** **(1.13–1.41)**	**1.17** **(1.11–1.23)**
	≥ 2 times (*N* = 414,193)	**1.23** **(1.16–1.29)**	1.07 (0.51–2.26)	0.96 (0.80–1.15)	**1.26** **(1.10–1.44)**	**1.59** **(1.37–1.85)**	**1.30** **(1.21–1.41)**	1.50 (0.62–3.60)	1.03 (0.81–1.32)	**1.27** **(1.05–1.54)**	**1.30** **(1.21–1.41)**
7%	1 time (*N* = 516,383)	**1.22** **(1.17–1.28)**	None	1.02 (0.83–1.24)	**1.25** **(1.14–1.37)**	**1.58** **(1.45–1.72)**	**1.22** **(1.14–1.30)**	None	0.88 (0.65–1.19)	**1.31** **(1.17–1.48)**	**1.22** **(1.14–1.30)**
	≥ 2 times (*N* = 115,333)	**1.48** **(1.36–1.61)**	None	1.07 (0.34–3.31)	1.18 (0.93–1.50)	**1.75** **(1.50–2.05)**	**1.53** **(1.35–1.74)**	None	0.90 (0.13–6.37)	**1.56** **(1.14–2.14)**	**1.53** **(1.35–1.74)**
10%	1 time (*N* = 183,968)	**1.35** **(1.26–1.44)**	None	None	1.02 (0.76–1.39)	**1.67** **(1.53–1.82)**	**1.36** **(1.24–1.49)**	None	None	1.19 (0.81–1.76)	**1.36** **(1.24–1.49)**
	≥ 2 times (*N* = 21,385)	**2.04** **(1.72–2.42)**	None	None	None	**2.48** **(2.08–2.95)**	**1.91** **(1.49–2.45)**	None	None	None	**1.91** **(1.49–2.45)**

BMI, body mass index; BW, body weight; VIM, variability independent of the mean; Q, quartile

Bolds are statistically significant

### Subgroup analyses

Online-only Table 1 shows the association between the changes in BW and dementia risk according to sex. Overall, males tended to have a higher risk of changes in BW. Online-only Table 2 presents the associations between BW change and dementia risk in accordance with personal medical history of HTN and DM. The subgroups were divided into those without HTN or DM and those with both HTN and DM. Overall, those with both HTN and DM had a higher risk of developing dementia than those without HTN and DM.

## Discussion

In this large-cohort dataset and a 9.67-year follow-up, the highest quartile of VIM was linked to an increased incidence of dementia. The newly introduced concept of the BW cycle, which focuses on the direction of weight change, showed an even higher risk of dementia when combined with VIM. Specifically, individuals in the highest VIM quartile had a 1.52-fold higher risk of dementia than those in the lowest quartile. Moreover, a 5% BW cycle with bidirectional changes generated a hazard ratio of 1.56. Notably, a 10% BW cycle with 2 changes within the highest quartile of VIM was associated with a 2.368-fold increase in dementia risk. The average age at dementia diagnosis was 66.23 years, which is lower than that reported in previous studies [17, 18]. These findings suggest that, besides the range of variability, the direction of variability may be a significant factor.

BW variability can be driven by physical activity, intentional weight loss, and dietary factors, which may cause morbidities such as metabolic syndrome, cardiovascular disease, and cerebrovascular disease [7]. Significant BW fluctuation impaired glucose tolerance and insulin resistance, and thereby potentially accelerated atherogenesis [3, 12, 19]. Insulin resistance in adipocytes can impair central insulin action, which is involved in cognitive processes and energy and glucose homeostasis in the brain, and could thereby increase vulnerability to amyloid toxicity and oxidative stress [20]. An association of mid-life-BW variability with increased risk of dementia in later years has been established [4, 21, 22]. Our study aligns with prior findings on the impact of mid-life-BW variability on dementia risk, and highlights that the direction of BW changes, rather than merely the variability size, is more significantly associated with the earlier onset of dementia. The lower BMI group with a higher BW cycle had a higher risk of dementia. Obesity is a well-known risk factor for dementia. However, paradoxically, low BMI may increase the risk of dementia [9, 18]. One study showed that patients with a low BMI have a reduced mesial temporal cortex volume [23], and those results clarify why our results showed an increased risk of dementia in the lower BMI group with a higher BW cycle.

Of the numerous studies on BW variability and parameters to calculate BW variability, most have mainly focused on investigating the effect of the magnitude of variability and have not considered the aspects of BW variability whereby the same magnitude of BW variability may pose different levels of health risk [7, 20]. The results of this study clearly demonstrated that, even among the participants with high BW variability (highest quartile of VIM), the risk of dementia increased in a dose–response manner with higher levels and cycles of BW. Further research to evaluate whether changes in body composition or waist circumference accompany fluctuations in the BW cycle is needed.

Our study is limited in some points. We did not include individuals who underwent insufficient health screening, despite the complete enumeration approach. This strategy excluded populations who may present a higher risk than the participants, as it may neglect or overestimate the result [24, 25]. However, the proportion of individuals undergoing health screening in Korea was expected to increase to 74.1% by 2021 [26]. We believe that our study may sufficiently representative of the Korean population. Besides, our study is limited in that we did not perform subgroup analyses to examine the impact of different types of dementia on the observed weight changes, and thus did not consider the potential influence of dementia subtype. Previous studies have shown that patients with DM are at higher risk of developing vascular dementia, as evidenced by BW changes [27], while the general population is at relatively higher risk of developing Alzheimer’s dementia [4]. Future studies that analyze specific types dementia in more detail may shed light on the trends observed in the various population regarding associations with dementia subtypes. Furthermore, the failure to include education level of participants in the analysis due to lack of data may also be a limitation of our study. Given that education may be an important factor in cognitive function [28], future research should include the aspect of the study. Another limitation is that the direction of BW increase or decrease was not assessed in this study. Since weight loss may be associated with cancer [29], future studies should be done with more details, such as excluding cancer comorbidities. The next limitation is that dementia was defined based on the ICD-10 code, which, owing to the nature of the claims data, might induced some overestimation. However, because dementia occurs irrespective of the BW cycle and VIM, this aspect should not be a concern. Finally, this study was performed within a homogeneous ethnic population; thus, an extended study with broader populations should be conducted to verify these findings.

Nevertheless, our study has important implications for the utilization of comprehensive and representative datasets. The reliance of the study on this extensive, whole-population data enhances the robustness and generalizability of the findings, which provide an accurate reflection of the broader population and strengthen the validity of the results. Furthermore, this study introduces a novel and conceptual approach to assess BT variability by differentiating the BW cycle within the VIM and, moreover, demonstrates that these parameters could be more effectively used complementarily as BW variability indices. Compared with previous studies on dementia and midlife BW variability, we revealed that a higher BW cycle, when combined with BW variability (VIM), could be associated with the earlier onset of dementia.

## Conclusion

The BW cycle, defined as either an upward or a downward direction of the BW, potentially constitutes a more effective index when combined with BW variability (VIM). These combined approaches predicted a higher risk of early-onset of dementia than prediction with VIM alone.

### Electronic supplementary material

Below is the link to the electronic supplementary material.

Supplementary Material 1

## Data Availability

No datasets were generated or analysed during the current study.
