# Supplementary material for: Association of midlife body-weight variability and cycles with earlier dementia onset: a nationwide cohort study
Source: Alzheimers Res Ther. 2024 Apr 25;16:91. doi: 10.1186/s13195-024-01460-5 (PMC11044324; doi:10.1186/s13195-024-01460-5)
Supplement: Supplementary file 1 — Supplementary Material 1 [file 13195_2024_1460_MOESM1_ESM.docx]

**Online-only Table 1. The sex-stratified combined effect of the associations of BW-VIM and BW cycle with incident dementia**

|  | | **Male participants**  **(N=2,412,975)** | | | | | **Female participants**  **(N=1,223,013)** | | | | |
| --- | --- | --- | --- | --- | --- | --- | --- | --- | --- | --- | --- |
|  | | **BW cycle only** | **VIM Q1** | **VIM Q2** | **VIM Q3** | **VIM Q4** | **BW cycle only** | **VIM Q2** | **VIM Q1** | **VIM Q2** | **VIM Q3** |
| VIM only | | – | 1.0 (ref) | **1.09 (1.03**–**1.15)** | **1.26 (1.19**–**1.33)** | **1.58 (1.49**–**1.66)** | – | 1.0 (ref) | **1.06 (1.00–1.12)** | **1.19 (1.12–1.26)** | **1.44 (1.36–1.52)** |
| <3% | | 1.0 (ref) | 1.0 (ref) | **1.14 (1.04**–**1.24)** | **1.14 (1.03**–**1.26)** | **1.49 (1.36**–**1.64)** | 1.0 (ref) | 1.0 (ref) | 1.06 (0.97–1.17) | **1.13 (1.02–1.24)** | **1.40 (1.28–1.53)** |
| 3% | 1 time | **1.10 (1.05–1.15)** | 1.07 (0.98–1.18 | 1.09 (0.98–1.20) | **1.31 (1.17**–**1.46)** | **1.41 (1.24**–**1.61)** | 1.04 (0.99–1.09) | 0.97 (0.88–1.07) | 0.96 (0.86–1.06) | **1.13 (1.00–1.26)** | **1.34 (1.19–1.52)** |
|  | ≥2 times | **1.14 (1.09–1.20)** | 0.88 (0.77–1.01) | **1.13 (1.01**–**1.26)** | **1.36 (1.20**–**1.54)** | **1.40 (1.21**–**1.62)** | **1.09 (1.04–1.14)** | 0.97 (0.83–1.13) | 1.11 (0.98–1.25) | **1.16 (1.01–1.33)** | **1.26 (1.08–1.46)** |
| 5% | 1 time | **1.16 (1.11–1.21)** | 1.00 (0.82–1.21 | **1.09 (1.00**–**1.20)** | **1.26 (1.15**–**1.40** | **1.57 (1.40**–**1.77)** | **1.14 (1.10–1.20)** | 0.75 (0.55–1.03) | 1.08 (0.98–1.19) | **1.25 (1.14–1.38)** | **1.40 (1.25–1.57)** |
|  | ≥2 times | **1.25 (1.18–1.33)** | 0.86 (0.39–1.93) | 0.93 (0.77–1.12) | **1.21 (1.03**–**1.41)** | **1.57 (1.32**–**1.87)** | **1.22 (1.14–1.30)** | 1.99 (0.89–4.44) | 1.05 (0.84–1.32) | **1.28 (1.09–1.50)** | **1.51 (1.26–1.79)** |
| 7% | 1 time | **1.25 (1.18–1.31)** | None | 1.03 (0.85–1.26) | **1.36 (1.23**–**1.50)** | **1.51 (1.36**–**1.68)** | **1.17 (1.11–1.24)** | None | 0.84 (0.61–1.14) | **1.13 (1.01–1.26)** | **1.41 (1.27–1.55)** |
|  | ≥2 times | **1.64 (1.50–1.80)** | None | 1.48 (0.56–3.96) | **1.41 (1.12**–**1.78)** | **1.85 (1.54**–**2.21)** | **1.27 (1.13–1.42)** | None | None | 1.07 (0.76–1.48) | **1.48 (1.22–1.80)** |
| 10% | 1 time | **1.41 (1.31–1.52)** | None | None | 1.13 (0.84–1.51) | **1.75 (1.59**–**1.92)** | **1.27 (1.17–1.38)** | None | None | 0.98 (0.65– 1.50) | **1.47 (1.33–1.62)** |
|  | ≥2 times | **2.14 (1.79–2.56)** | None | None | None | **2.60 (2.16**–**3.13)** | **1.72 (1.37–2.16)** | None | None | None | **1.97 (1.56–2.48)** |

BW, body weight; VIM, variability independent of the mean; Q, Quartile

**^†^**Adjusted for age, sex, body mass index (BMI), systolic blood pressure (SBP), diastolic blood pressure (DBP), total cholesterol, estimated glomerular filtration rate (eGFR), health-related behaviors (smoking status, alcohol intake, and physical activity), medical history (hypertension, type 2 diabetes mellitus, heart disease, and stroke), and family history (hypertension, type 2 diabetes mellitus, heart disease, and stroke).

**^‡^**Bolds are statistically significant.

**Online-only Table 2. The combined effect of the associations of BW-VIM and BW cycle with incident dementia stratified by medical history of hypertension and type 2 diabetes mellitus**

|  | | **Without HTN and DM**  **(N=1,354,168)** | | | | | **With HTN and DM**  **(N=2,281,820)** | | | | |
| --- | --- | --- | --- | --- | --- | --- | --- | --- | --- | --- | --- |
|  | | **BW cycle only** | **VIM Q1** | **VIM Q2** | **VIM Q3** | **VIM Q4** | **BW cycle only** | **VIM Q2** | **VIM Q1** | **VIM Q2** | **VIM Q3** |
| VIM only | | – | 1.0 (ref) | **1.07 (1.02–1.13)** | **1.26 (1.21–1.32)** | **1.55 (1.48–1.62)** | – | 1.0 (ref) | **1.11 (1.02–1.21)** | **1.17 (1.08–1.27)** | **1.52 (1.40–1.64)** |
| <3% | | 1.0 (ref) | 1.0 (ref) | **1.42 (1.24–1.62)** | 0.99 (0.86–1.13) | 1.03 (0.89–1.18) | 1.0 (ref) | 1.0 (ref) | **1.52 (1.42–1.64)** | 1.05 (0.97–1.14) | 1.04 (0.96–1.13) |
| 3% | 1 time | **1.09 (1.05–1.13)** | 1.12 (0.95–1.33) | 1.19 (0.98–1.45) | 0.88 (0.71–1.08) | 0.12 (0.95–1.32) | 1.02 (0.96–1.10) | **1.28 (1.17–1.41)** | **1.51 (1.37–1.67)** | 0.96 (0.85–1.07) | **1.15 (1.05–1.27)** |
|  | ≥2 times | **1.15 (1.11–1.20)** | **1.27 (1.05–1.53)** | **1.47 (1.19–1.81)** | 0.69 (0.47–1.01) | 1.10 (0.96–1.26) | 1.07 (0.99–1.14) | **1.31 (1.18–1.46)** | **1.37 (1.21–1.54)** | 1.00 (0.83–1.19) | **1.11 (1.02–1.20)** |
| 5% | 1 time | **1.17 (1.13–1.21)** | **1.26 (1.10–1.44)** | **1.54 (1.30–1.81)** | 1.22 (0.39–3.81) | 0.92 (0.67–1.24) | **1.13 (1.06–1.21)** | **1.30 (1.20–1.41)** | **1.54 (1.40–1.69)** | 1.23 (0.64–2.36) | 1.02 (0.86–1.20) |
|  | ≥2 times | **1.29 (1.22–1.35)** | 1.22 (0.97–1.53) | 1.13 (0.84–1.53) | None | 0.88 (0.61–1.26) | **1.45 (1.04–1.26)** | **1.30 (1.14–1.47)** | **1.75 (1.53–2.01)** | None | 1.02 (0.85–1.23) |
| 7% | 1 time | **1.25 (1.19–1.30)** | None | **1.53 (1.32–1.76)** | None | 0.93 (0.59–1.46) | **1.15 (1.07–1.25)** | None | **1.51 (1.39–1.64)** | 1.43 (0.54–3.81) | **1.44 (1.17–1.77)** |
|  | ≥2 times | **1.55 (1.43–1.67)** | None | **1.49 (1.09–1.96)** | None | 0.81 (0.44–1.46) | **1.36 (1.16–1.60)** | None | **1.82 (1.57–2.11)** | None | 1.18 (0.91–1.54) |
| 10% | 1 time | **1.36 (1.28–1.45)** | None | None | None | **2.48 (1.18–3.40)** | **1.38 (1.23–1.56)** | None | None | None | **2.44 (2.07–2.87)** |
|  | ≥2 times | **1.99 (1.70–2.33)** | None | None | None | **1.44 (1.32–1.56)** | **2.18 (1.60–3.00)** | None | None | None | **1.37 (1.30–1.43)** |

BW, body weight; VIM, variability independent of the mean; Q, quartile

**^†^**Adjusted for age, sex, body mass index (BMI), systolic blood pressure (SBP), diastolic blood pressure (DBP), total cholesterol, estimated glomerular filtration rate (eGFR), health-related behaviors (smoking status, alcohol intake, and physical activity), medical history (hypertension, type 2 diabetes mellitus, heart disease, and stroke), and family history (hypertension, type 2 diabetes mellitus, heart disease, and stroke).

**^‡^**Bolds are statistically significant.
